# Design and rationale for the life after stopping tyrosine kinase inhibitors (LAST) study, a prospective, single-group longitudinal study in patients with chronic myeloid leukemia

**DOI:** 10.1186/s12885-018-4273-1

**Published:** 2018-04-02

**Authors:** Ehab Atallah, Charles A. Schiffer, Kevin P. Weinfurt, Mei-Jie Zhang, Jerald P. Radich, Vivian G. Oehler, Javier Pinilla-Ibarz, Michael W. N. Deininger, Li Lin, Richard A. Larson, Michael J. Mauro, Joseph O. Moore, Ellen K. Ritchie, Neil P. Shah, Richard T. Silver, Martha Wadleigh, Jorge Cortes, James Thompson, Jessica Guhl, Mary M. Horowitz, Kathryn E. Flynn

**Affiliations:** 1 Department of Medicine, Medical College of Wisconsin, 9200 W Wisconsin Ave, Milwaukee, WI 53226 USA; 2 Karmanos Cancer Institute, Wayne State University School of Medicine, Detroit, MI USA; 3 Department of Population Health Sciences, Duke University School of Medicine, Durham, NC USA; 4 Division of Biostatistics, Medical College of Wisconsin, Milwaukee, WI USA; 5 Clinical Research Division, Fred Hutchinson Cancer Research Center, Seattle, WA USA; 6 Department of Malignant Hematology, H. Lee Moffitt Cancer Center & Research Institute, Tampa, FL USA; 7 Division of Hematology and Hematologic Malignancies, Huntsman Cancer Institute, The University of Utah, Salt Lake City, UT USA; 8 Department of Medicine and Comprehensive Cancer Center, University of Chicago, Chicago, IL USA; 9 Memorial Sloan Kettering Cancer Center, New York, NY USA; 10 Duke Cancer Institute, Durham, NC USA; 11 Weill Medical College of Cornell University, New York, NY USA; 12 Department of Medicine, University of California at San Francisco, San Francisco, CA USA; 13 Department of Medical Oncology, Dana-Farber Cancer Institute, Boston, MA USA; 14 University of Texas MD Anderson Cancer Center, Houston, TX USA; 15 Roswell Park Cancer Institute, Buffalo, NY USA; 16 Cancer Center, Medical College of Wisconsin, Milwaukee, WI USA

**Keywords:** Chronic myeloid leukemia, Oncology, Targeted therapy, Tyrosine kinase inhibitor, Discontinuation, Clinical trial, Study design, Patient-reported outcome

## Abstract

**Background:**

Treatment of chronic myeloid leukemia with a tyrosine kinase inhibitor (TKI) offers significant improvements over previous treatments in terms of survival and toxicity yet nevertheless is associated with reduced health-related quality of life and very high cost. Several small studies from Europe and Australia suggested that discontinuing TKIs with regular monitoring was safe.

**Methods:**

The Life After Stopping TKIs (LAST) study is a large, U.S.-based study that aims to improve the evidence for clinical decision making regarding TKI discontinuation with monitoring in patients with chronic myeloid leukemia who have a deep molecular response to TKI therapy. The LAST study is a non-randomized, prospective, single-group longitudinal study of 173 patients. The co-primary objectives are to determine the proportion of patients who develop molecular recurrence (> 0.1% BCR-ABL^IS^) after discontinuing one of four TKIs (imatinib, dasatinib, nilotinib, or bosutinib) and to compare the patient-reported health status of patients before and after stopping TKIs. Outcomes are assessed at baseline and throughout the 36-month study follow-up period with a central laboratory used for blood samples. All samples with undetectable BCR-ABL are also examined using digital polymerase chain reaction, which is a more sensitive nanofluidic polymerase chain reaction system.

**Discussion:**

Because of their high cost and side effects, discontinuation of TKIs for patients with chronic myeloid leukemia who have a deep molecular response to TKI therapy is a promising approach to treatment. The LAST study is the largest U.S.-based TKI discontinuation study. It is the first to allow participation from patients on any of 4 first- and second-generation TKIs, includes a robust approach to measurement of clinical and patient-reported outcomes, and is using digital polymerase chain reaction to explore better prediction of safe discontinuation.

**Trial registration:**

This study was registered prospectively on October 21, 2014 and assigned trial number NCT02269267.

## Background

Tyrosine kinase inhibitors (TKIs) have dramatically improved the survival of patients with chronic myeloid leukemia (CML), leading to an unforeseen increase in prevalence. Prevalence in 2010 was estimated at more than 70,000 patients in the U.S. and is projected to rise to 180,000 by 2050 [1]. Current guidelines recommend continuation of TKI therapy indefinitely [2] (www.nccn.org). TKIs offer a significant improvement over previous CML treatments in terms of survival and toxicity [3, 4], yet nevertheless are associated with reduced health status, including fatigue, nausea, depression, sleep disturbances, diarrhea, pain, fluid retention, and skin problems [5], especially as compared to peers without cancer [6]. With only 15 years of follow up, the very long-term side effects of TKIs remain unknown [7]. Of note, some deleterious side effects have been recognized with longer follow-up, for example, pulmonary hypertension in patients on dasatinib [8] and peripheral arterial occlusive disease in patients on nilotinib [9]. Finally, TKI therapies are among the most expensive, costing $92,000–138,000 per patient annually (in 2013 dollars), placing a financial burden on the U.S. health care system as well as on individual patients and their families [10].

Initially several small studies from Europe and Australia suggested that discontinuing TKIs with regular monitoring is safe [11–16]. In these studies, 22–61% of patients with CML in a TKI-induced complete molecular response maintained this response after discontinuation of TKIs, and all patients with recurrent CML responded to reintroduction of TKI therapy. These studies have varied with regard to sample size (40–124 at the time LAST was funded), definition of recurrence, and duration and type of TKI therapy before discontinuation. Taken together, these results were compelling; however, CML experts agreed that too little was known about the variables governing maintenance of molecular response versus recurrence of leukemia to recommend TKI discontinuation with monitoring for routine clinical practice [11, 17, 18]. More recently, several large studies have completed accrual and additional long term results are pending [19–22]. Finally, little was known about the impact of discontinuation on health-related quality of life, leaving patients and providers without critical information for deciding whether to discontinue TKI therapy.

The National Cancer Institute funded the Life After Stopping TKIs (LAST) study to improve the evidence for clinical decision making regarding TKI discontinuation with monitoring in CML patients and to shift clinical practice paradigms by reducing uncertainty regarding TKI discontinuation, and providing critical information about patient health-related quality of life. The study has completed the planned accrual, and all subjects are now being monitored. In this manuscript we describe the design and rationale for the LAST study.

## Methods/design

The co-primary objectives of the LAST study are to determine the proportion of patients with CML who develop molecular recurrence (> 0.1% BCR-ABL^IS^) after discontinuing TKIs and to compare the patient-reported health status of patients before and after stopping TKIs. The LAST study will also analyze whether there are disease, patient-related, or treatment-related factors that predict molecular recurrence, develop a risk scoring system to predict the patient’s risk of molecular recurrence after stopping TKI, assess whether specific time points are more important for clinical prediction of recurrence and develop an optimal follow up schedule, and describe the patient-reported health status of patients who resume TKI therapy after molecular recurrence. Exposure to TKIs and patient-reported outcomes (PROs) vary between patients, so patients serve as their own comparators in assessing time to recurrence and health status on and off TKI therapy. We will compare across patients to determine predictors of recurrence.

### Participants

The target enrollment was 173 patients who met the inclusion and exclusion criteria listed in Table 1. Patients were enrolled at 14 academic medical centers.

**Table 1:** LAST Study Inclusion and Exclusion Criteria

Inclusion Criteria
Age 18 or older
Willing and able to give informed consent
Diagnosed with CML in chronic phase and have either the b3a2 (e14a2) or b2a2 (e13a2) variants that give rise to the p210 BCR-ABL protein
Currently taking imatinib, dasatinib, nilotinib or bosutinib
Has been on TKI therapy for at least 3 years
Documented BCR-ABL < 0.01% or undetectable BCR-ABL by RQ-PCR for at least 2 years according to the patient’s local lab
Documented BCR-ABL < 0.01% or undetectable BCR-ABL by RQ-PCR at least 3 times prior to screening according to the patient’s local lab
Two (2) Screening PCRs have been completed and both results are < 0.01% (better than MR4, i.e,. > 4 log reduction) by central lab
Has been on any number of TKIs, but has not been resistant to any TKI (changes made for intolerance are allowed)
Has been compliant with therapy per treating physician
Exclusion Criteria
Prior hematopoietic stem cell transplantation
Poor compliance with taking TKI
Unable to comply with lab appointments schedule and PRO assessments
Life expectancy less than 36 months
Resistant to previous TKI therapy
Pregnant or lactating women

### Screening and enrollment

A paper log of all potential patients has been kept by each site, including individuals who decide not to participate in or who are found to be ineligible for the study. Screening is performed for potential study patients after they have consented to trial participation. After informed consent, patients are assessed by RQ-PCR by the Central Molecular Diagnostics Core Laboratory at the Fred Hutchinson Cancer Center (Central Lab) twice, at least 21 days apart, to confirm that the BCR-ABL is < 0.01% (better than MR4 i.e. > 4 log reduction). PROs were collected twice during the screening period to record patients’ baseline, on-therapy health status. The coordinating center reviewed all documents to confirm eligibility. Patients were considered to be enrolled in the study once the coordinating site has confirmed that all screening eligibility criteria had been met; the TKI was stopped within 7 days of enrollment.

### Monitoring

Regular monitoring of BCR-ABL assessments proceeds as follows: if patient was in the first 6 months of study, RQ-PCR is performed monthly; if in month 7–24, RQ-PCR is performed every 2 months; and if in third year, RQ-PCR is performed every 3 months. PROs are assessed monthly for the first 6 months, an additional assessment at 8 months, and then every 6 months until study end. For patients who restart TKI therapy, RQ-PCR is performed approximately every 3 months at the Central Lab until the patient’s BCR-ABL is < 0.01% (MR^4^) two consecutive times or for the duration of the study, whichever comes first. PROs are assessed in these patients approximately every 3 months for 1 year and then approximately every 6 months until study end. Clinical data is recorded by study coordinators using OnCore CTMS; PRO data is recorded by patients using REDCap.

### Assessment of BCR-ABL1

Enrolled patients have blood draws for RQ-PCR at the participating site laboratory, though with permission they can have certain labs drawn by other laboratories closer to home. All peripheral blood samples are shipped fresh to the Central Lab to perform RQ-PCR and digital testing. The Central Lab uses standard molecular RQ-PCR monitoring in all patients on the International Scale (IS). All samples with undetectable BCR-ABL are also examined using digital PCR, which is a more sensitive nanofluidic PCR system (Fluidigm Corporation, South San Francisco, CA) with an increased sensitivity of > 2 log beyond the standard PCR assay [23, 24]. Digital PCR follows the same schedule as RQ-PCR monitoring unless CML recurs, then only RQ-PCR testing is used thereafter. Digital PCR results will be used for research questions only, with results available to clinicians only upon conclusion of the study. Thus therapy decisions are made based on RQ-PCR only as per existing treatment guidelines. During the monitoring phase, if BCR-ABL is ≥0.01% for the first time, then PCR testing is repeated monthly for 3 months. If BCR-ABL is ≥0.1% at any time, patients are instructed to restart TKI therapy. The decision of which TKI to restart is left to the patient and his/her physician.

### Assessment of PROs

PRO assessments are primarily administered electronically at the clinic/lab, with the site coordinator meeting with the patient at the time of blood draws and administering the assessment through the secure REDCap platform on an iPad. For participants who do not complete their PRO assessment at the clinic, the local site coordinator can 1) email the REDCap link to the participant to complete the assessment online, 2) access the assessment in REDCap and read the quesitons to the participant over the telephone, reporting the participant’s responses directly into REDCap, or 3) give the participant a paper version of the assessment. The selection of the PRO domains being measured was based on literature review, including a recent high-quality qualitative study on health-related quality of life in 137 CML patents on TKIs [25]. We used PROMIS measures when available (Table 2). PROMIS measures are scored on a standardized scale, where 50 corresponds to the average in the general U.S. population with a standard deviation of 10. For additional CML-specific symptoms, we used the EORTC QLQ-CML Symptom Burden scale, which includes single items on abdominal pain, dry mouth, skin problems, headaches, joint pain or swelling, eye problems, etc. [26] In a longer baseline assessment, we collected sociodemographic characteristics and monthly out of pocket expenses for TKIs. In the 3 month assessment we asked additional questions about medications.

**Table 2:** LAST Study Measurement of Patient-Reported Outcomes

Measure	# Items
PROMIS Fatigue^a^	CAT
PROMIS Depression^a^	CAT
PROMIS Sleep Disturbance^a^	CAT
PROMIS GI Symptoms - Diarrhea^a^	2
PROMIS GI Symptoms - Nausea	1
PROMIS Physical Function	CAT
PROMIS Anxiety	CAT
PROMIS Sleep Related Impairment	CAT
PROMIS Pain Interference	CAT
PROMIS Pain Intensity	1
PROMIS Ability to Participate in Social Roles and Activities	CAT
PROMIS Social Isolation	CAT
PROMIS Applied Cognition General	CAT
PROMIS Interest in Sexual Activity	1
PROMIS Satisfaction with Sex Life	1
PROMIS Alcohol Use	2
PROMIS Global Self-Rated Health	1
PROMIS Global Quality of Life	1
EORTC CML Symptom Burden (includes 1 item on musculoskeletal pain^a^)	13
Ability to work	2
Ability to pay monthly bills	1
Changes in use of pharmaceuticals	6

*Abbreviations*: *EORTC* European Organisation for Research and Treatment of Cancer; *CAT* Computerized Adaptive Test; *GI* Gastrointestinal; *PROMIS* Patient-Reported Outcomes Measurement Information System

^a^Hypothesized change of ≥0.3 standardized effect size

### Analyses

We will use a 2-tailed significance level of α = 0.05 for all assessments. Statistical analyses will be conducted using SAS (SAS Institute, Inc).

#### Required sample size

We needed sufficient power to detect differences by patient characteristics in order to predict CML recurrence. With 173 patients and assuming 5% loss to follow-up and a 2-tailed significance level of α = 0.05, we will have 90% power to detect a difference of 25% between groups of equal size (1:1 ratio) in relapse-free survival (RFS) at 18 months.

We also needed sufficient power to detect the smallest policy-relevant change in health status, which we estimate as an effect size of 0.3, i.e., corresponding to about 1/3 of a standard deviation. We used simulation methods to conduct a power analysis for a piecewise linear mixed-effects model in SAS 9.3, assuming 10% missing data per year to account for dropout and missed assessments. Relative to the null hypothesis of no time effect (that is, no difference in PROs over time with TKI discontinuation and reintroduction), a sample size of 173 patients provides > 90% power to detect a change of 0.3 and > 85% power to detect an effect size of 0.25.

#### Analysis plan for clinical endpoints

The clinical outcomes to be examined in this proposal include the primary event of CML molecular recurrence (opposite relapse-free survival [RFS]) and death in complete remission (DCR). For the univariate analysis the probabilities of RFS will be calculated using the KaplanMeier- estimator. Probabilities of molecular recurrence and DCR will be generated using cumulative incidence estimates to account for competing risks. Cox proportional hazards model [27] and Fine and Gray’s subdistribution hazards model [28] will be used to determine the effect of clinical characteristics on RFS and CML recurrence after TKI discontinuation, respectively. The baseline clinical risk factors that will be considered in regression analyses include sex, age, type of TKI, time to MR 4.5, duration in MR 4.5, Sokal Risk score at diagnosis, and BCR-ABL1 transcript levels measured by digital PCR. The following analysis plan will be implemented.

First, for the continuous variables, including time to initial MR 4.5, a martingale residual plot will be applied to evaluate the potential threshold cut point(s) for the effect on RFS and the maximum partial likelihood method will be used to identify optimal cut point(s). Second, univariate probability of RFS, molecular relapse, and DCR with 95% confidence interval (CI) will be computed by each clinical risk factor. Third, a Cox regression model building procedure will be used to identity significant risk factors associated with molecular recurrence. The assumption of proportional hazards for each factor in the Cox model will be tested using time-dependent covariates. When the test indicates differential effects over time (non-proportional hazards), models will be constructed breaking the post-stopping TKI time course into two periods, using the maximized partial likelihood method to find the most appropriate breaking time point. Following this, the proportionality assumptions will be tested again. Factors that are significant at a 5% level will be kept in the final model. The potential interactions between all significant risk factors will be tested. Fourth, based on the final Cox model, a risk scoring system will be developed to predict the patient’s risk of molecular recurrence after stopping TKI. A 3–4 level scoring system will be considered as appropriate for the data. Our sample is sufficient to generate the risk scoring system, but future studies will be necessary for an independent validation of the system. Fifth, we will assess whether specific time points are more important for clinical prediction within this schedule. At each pre-scheduled follow-up time, we will calculate the recurrence rate and treatment failure rate (recurrence or DCR) with its 95% CI. We will use the log transfer approach [29] to calculate the CI to force the CI to be within proper range (0, 1). We will make recommendations for the optimal follow-up schedule based on the estimated recurrence rates at each time point.

#### Analysis plan for PROs

The primary objective of the analysis of PRO measures in this study is to describe what happens to patients’ health status after stopping TKI therapy. A secondary objective is to describe what happens to the health status of patients who resume TKI therapy after CML recurrence. While we originally proposed a single piecewise model to answer both questions, this approach includes the people who did not relapse in the model to answer the 2nd question. Thus, we pre-specified a revised approach that uses separate models for each of the two objectives (outlined in further detail below).

We plan to analyze the components of health-related quality of life separately (that is, depression separate from fatigue separate from GI symptoms, etc), since combining them into a summary score can dilute the effects of the individual (and not necessarily related) components and thus mask true change. However, this “battery approach” to PRO assessment has the drawback of resulting in multiple, individual component scores and 1) raises the possibility of obtaining conflicting results of the different components and 2) creates a potential multiple comparisons problem for statistical testing. Pre-specifying the major expected relationships and corresponding statistical comparisons minimizes such potential problems; this is the approach we have taken in this study.

We hypothesize that, following TKI discontinuation, fatigue, depression, sleep disturbance, and diarrhea will improve by at least 3 points each by 6 months post-discontinuation (corresponding to the standardized effect size of 0.3 used in our sample size estimation). Since the initiation of the LAST study, a syndrome of musculoskeletal pain has been reported to occur in some CML patients after discontinuing TKI therapy [30]. Thus, this will be an outcome of special interest. Trajectories of the remaining PROs will also be described, but we have no a priori hypotheses about how they will change. Likewise, we hypothesize worsening in fatigue, depression sleep disturbance, and diarrhea by at least 3 points each by 6 months post-resumption of TKI therapy. In reporting all results, we will use 95% confidence intervals and graphical presentations wherever possible to convey the uncertainty associated with our findings.

Our objectives will be pursued in the context of piecewise longitudinal mixed-effects models for each of the PRO endpoints. This modeling approach offers several advantages: the likelihood-based estimation means that all available data from each patient are used; correlations within patients over time are addressed, and any missing data can be considered ignorable conditional upon the observed data [31].

To describe what happens to patients’ health status after stopping TKI therapy, all patient data while off TKI therapy will be included. For each PRO domain, growth curve models will be fit to the data with actual time after enrollment added to the model as a fixed effect (treated as a continuous variable drawn from data collection dates). Because side effects associated with TKIs are expected to diminish in 1–3 months after discontinuation of TKIs, the change rate in PROs is not likely to be consistent through the 3-year study period. Although the growth curves are not expected to be linear, it is reasonable to approximate the curves to lines over short intervals. Linear approximation will facilitate model interpretation in a clinical meaningful way. Data visualizations and polynomial models will be used to determine suitable knots or pieces, that is, how many cut-points to include and where they should be located in time. New time variables will be derived to shape the growth curves after the number and location of knots are determined. Within-patient dependency will be addressed in the model via a random intercept and random slopes of each linear piece.

To describe health status changes after restarting TKIs, post recurrence, we will use the same methods described above with a few modifications. Data will be limited to the patients who had recurrence and restarted TKI treatment. A piecewise linear mixed-effects model will be used to separate each person’s trajectory into two pieces—one prior to restarting a TKI and one following the resumption of TKI. The main parameter of interest will be the estimated change from the new “baseline” at the assessment just prior to restarting TKI and the 3 month assessment.

For PROMIS CATs, responses were required so there is no item-level missing. For missing data at the item level on other measures, we will handle missing values in each domain using the following approach. If at least 50% of the items per domain were answered, then we will adjust the score to ([Raw sum x number of items in the domain] / number of items answered). If fewer than 50% of the items in a domain were answered, we will treat the domain as missing. We have chosen the longitudinal mixed effects model to allow the inclusion of all cases in our analyses, even those with missing values [32].

### Ethics review

The Medical College of Wisconsin (MCW) institutional review board (IRB) offered to be the IRB of record for all participating sites, but no sites agreed to cede regulatory responsibility. After the MCW IRB granted initial approval, the multi-site program staff provided regulatory documents to the participating institutions including initial approval documents, consent forms, site initiation forms, and amendments. Each participating institution then sought and obtained local IRB approval prior to activation of the study at their site. We allowed participating institutions to make minor changes to the consent form to reflect their institutional standards, and all site-approved consent forms and regulatory documents were re-reviewed by the multi-site program staff at MCW. Site IRB approval was forwarded to the MCW IRB and sites were subsequently activated via a formal letter.

### Data safety monitoring committee

The MCW Cancer Center Data Safety Monitoring Committee is responsible for monitoring data quality and subject safety. A 6–8 member DSMC regularly reviews the protocol-specific data safety monitoring reports to provide recommendations on trial continuation, suspension, or termination. As the rate of disease progression to accelerated phase and/or blast crisis is about 1.2% per each year, the study will be put on hold if ≥3, 6, 9, 12, and 15 patients develop disease transformation at end of years 1, 2, 3, 4, and 5, cumulatively. In the case of a safety event suspending the study, a prompt cumulative examination of all data and circumstances of these events will be conducted to determine whether the study should be resumed, whether the protocol must be revised, or whether the study will be discontinued permanently.

### Rationale for study design

The LAST study is a non-randomized, prospective, single-group longitudinal study. Before choosing this design, we considered a randomized controlled trial (RCT) and a case-control study. With any study on this topic, enrollment is expected to be limited to patients who are both willing to stop TKI therapy and willing to participate in a research study. With that in mind, we identified the following concerns with a blinded RCT design: 1) compliance would be problematic—patients in the trial will know they are being closely monitored for recurrence, so they will have little incentive to take their assigned (blinded) pill if they were already inclined to stop TKI therapy; 2) blinding would be difficult, given the known side effects associated with TKIs; and 3) blinding would make it impossible to measure one of the potential adverse effects of TKI discontinuation that we are interested in, i.e., anxiety about disease recurrence after stopping. Our concerns with using a non-randomized control group for comparison in a case-control design are: 1) a comparison of recurrence between those who do or do not stop TKIs is unnecessary since we know that those who continue rarely recur and a proportion of those who stop will recur; and 2) we expect that patients who participate in this trial will have more severe side effects than those who are unwilling to stop their drug, and comparing these groups would likely overestimate changes in patient-reported outcomes. Within the single-group study design, we are comparing patients to themselves, which we believe should minimize this bias, but we will report this as a possible limitation when we interpret our results.

## Discussion

LAST is the largest US TKI discontinuation study to date. Most previous studies have included patients on a single TKI or required switching from imatinib to second generation TKI before discontinuation. LAST is the only study that allowed any of 4 TKIs. Like previous TKI discontinuation studies, LAST requires multiple years of TKI therapy before a discontinuation attempt. We chose to monitor for recurrence monthly for 6 months, then every 2 months until 24 months, and then every 3 months until 36 months to contribute to the overall picture of safety and provide recommendations for an optimal monitoring schedule. LAST also includes a robust approach to measurement, including central lab processing of blood samples during screening, discontinuation, and after restarting; a rigorous approach to assessment of patient-reported outcomes, and use of digital PCR. Accrual of 173 subjects was completed within 2 years at 14 participating study sites. No complications with the molecular monitoring in the Central Lab nor collection of PROs has occurred. All subjects are currently being monitored.
